# Clinical outcomes after assisted reproductive technology in twin pregnancies: chorionicity-based comparison

**DOI:** 10.1038/srep26869

**Published:** 2016-05-31

**Authors:** Luming Sun, Gang Zou, Xing Wei, Yan Chen, Jun Zhang, Nanette Okun, Tao Duan

**Affiliations:** 1 Fetal Medicine Unit & Prenatal Diagnosis Center, Department of Obstetrics, Shanghai First Maternity and Infant Hospital, Tongji University School of medicine, Shanghai, China; 2 Xinhua Hospital, Shanghai Jiao Tong University School of Medicine, Shanghai, China; 3 Maternal Fetal Medicine Program, Mt. Sinai Hospital, University of Toronto, Toronto, Ontario, Canada

## Abstract

The chorionicity–based evaluation of the perinatal risk in twin pregnancies after assisted reproductive technology (ART) is lacking. A retrospective review was performed of all twin pregnancies monitored prenatally and delivered at our hospital between 2010 and 2014. Chorionicity was diagnosed by ultrasound examination at first trimester and confirmed by postnatal pathology. Pregnancy and perinatal outcomes were prospectively recorded. Adjusted odds ratios (aOR) with 95% confidence intervals (CI) were calculated in a logistic regression model. A total of 1153 twin pregnancies were analyzed. The occurrence of preterm premature rupture of membranes (PPROM) was 3 times as frequent in monochorionic diamniotic (MCDA) twin pregnancies after ART as in those spontaneous counterparts (aOR 3.0; 95%CI 1.1–3.2). The prevalence of intrahepatic cholestasis of pregnancies (ICP) was significantly higher in dichorionic diamniotic (DCDA) twin pregnancies following ART compared to spontaneous DCDA pregnancies (aOR 3.3; 95%CI 1.3–5.6). Perinatal outcomes did not differ between two conception methods, either in MCDA or DCDA twin pregnancies. Based on differentiation of chorionicity, ART is associated with the increased risk of PPROM in MCDA twin pregnancies and with a higher rate of ICP in DCDA twin gestations. ART does not increase adversity of perinatal outcomes in twin pregnancies.

Assisted reproductive technology (ART) is currently the preferred management for various types of infertility, concomitant with increased rate of twin pregnancies. Recently, the related complication rates among ART versus spontaneous pregnancies have been explored^1^,^2^. An increasing body of evidence has demonstrated that twins born from ART-conceived pregnancies are at a higher risk of perinatal morbidity and mortality, such as birth defects and low birth weight, compared with twins from pregnancies conceived naturally^3^,^4^. The evidence on the impact of ART on maternal morbidities in twin pregnancies clarified by chorionicity is lacking. What exists is conflicting and mixed, with some studies showing a higher risk of such morbidities as gestational hypertension and gestational diabetes among ART twin pregnancies^5^,^6^,^7^, and others showing no impact of ART^8^,^9^,^10^. In addition, little has been reported on the impact of ART on maternal outcomes in monochorionic diamniotic (MCDA) twin pregnancies^5^. This lack of evidence may be due to the relative paucity of MCDA twin pregnancies after ART treatment^11^. Given the difference in perinatal risk between MCDA and dichorionic diamniotic (DCDA) twin pregnancies^12^,^13^,^14^, chorionicity-based analysis is thus necessary to assess the maternal risk in twin pregnancies resulting from assisted conception. The purpose of this study is to investigate selected clinical outcomes among ART–conceived twin pregnancies compared to spontaneously conceived twin pregnancies and to determine whether these outcomes are further affected by chorionicity.

## Results

**Figure 1:**
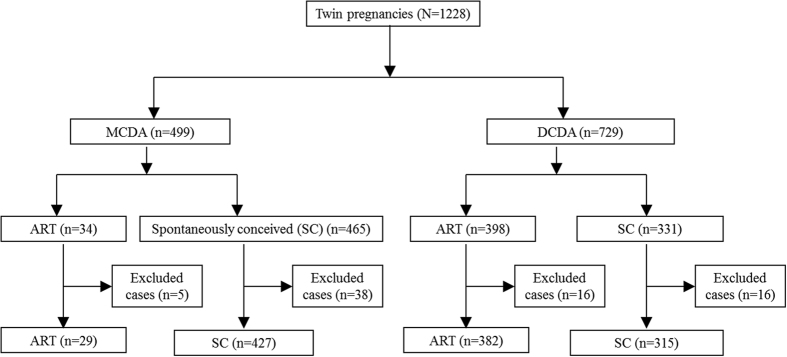
A flow chart to show the inclusion criteria in this study.

A total of 1228 twin pregnancies were monitored prenatally and delivered at our hospital during the study period. Structural anomalies were detected in 75 twin pregnancies and excluded from this study. The remaining 1153 twin pregnancies were analyzed. A flow chart illustrates the inclusion criteria of this study (Fig. 1).

Among MCDA twin pregnancies, the mean maternal age and body mass index were significantly higher in twin pregnancies following ART compared to spontaneously conceived twin pregnancies (Table 1). Among DCDA twin pregnancies, mean maternal age and the proportion of nullipara were significantly higher in twin pregnancies following ART compared to spontaneously conceived twin pregnancies. These maternal characteristics are selected as the independent variables in following multiple regression analysis due to their significant difference between compared subgroups and close association with pregnancy and perinatal outcomes. In addition, the interactions between independent variables was analyzed and displayed in Supplemental Table 1.

There was a higher risk of ICP among DCDA twins following ART compared to DCDA twins with spontaneous conceptions (aOR 3.3, 95% CI 1.3–5.6), and a higher risk of PPROM among MCDA twins following ART compared to MCDA twins with spontaneous conceptions (aOR 3.0, 95% CI 1.1–3.2). In the subgroup of MCDA twin pregnancies, the frequency of gestational hypertension (aOR 4.3, 95% CI .8–24.4) and placenta previa (aOR 4.5, 95% CI .8–24.6) tended to be higher in MCDA twins following ART compared to the counterparts conceived spontaneously, but this difference did not reach statistical significance. Chorionicity–based comparisons of maternal complications between twin pregnancies conceived spontaneously and following ART were displayed in Table 2. We further compared the rate of maternal complications between the subgroups of MCDA and DCDA twin pregnancies with control of conception models (Table 3). In the subgroup of twin pregnancies conceived spontaneously, the risk of maternal composite outcomes in MCDA twins was significantly increased compared to DCDA twins, 38.9% versus 52.7%, respectively (aOR 1.7, 95% CI 1.2–2.4). In the subgroup of twin pregnancies conceived following ART, the occurrence of maternal complications was comparable between MCDA and DCDA twins.

The perinatal outcomes did not significantly differ between MCDA twins from pregnancies conceived spontaneously or through ART. Similarly, no significant difference were found in perinatal outcomes between DCDA twins of spontaneous or ART conception. This data is summarized in Table 4.

This study also investigated the impact of chorionicity on perinatal outcome in the subgroups of twin pregnancies conceived spontaneously or after ART (Table 5). In both subgroups of twin pregnancies conceived spontaneously or after ART, the adversity of perinatal outcomes was significantly higher in MCDA twins compared to DCDA twins. The comparison of perinatal outcomes between MCDA and DCDA twins after excluding twin pregnancies with MCDA–specific complications is shown in Table 6. Delivery prior to 28 weeks was 7.7 to 14.4 times more likely to occur in MCDA twins compared to DCDA twins.

The prevalence of MCDA–specific perinatal complications by method of conception is displayed in Table 7. ART did not increase the risks of specific complications in MCDA twins, 24% (7/29) versus 39% (168/427), respectively (P = 0.11).

## Discussion

The present study demonstrates that ART is significantly associated with higher prevalence of PPROM in MCDA twin pregnancies following ART compared to MCDA twins with spontaneous conceptions and ICP in DCDA twin pregnancies following ART compared to DCDA twins with spontaneous conceptions. We also found ART does not increase perinatal morbidity outcomes in twin pregnancies of both types of chorionicity. MCDA twins remained at higher risks of adverse maternal and fetal outcomes compared to DCDA twins after TTTS, sIUGR, TAPS and TRAP cases were excluded, but not related to mode of conception.

Several studies have previously reported that ART-conceived pregnancies are independently associated with maternal complications^4^,^15^. However, data specific to ART conceived twin pregnancies are limited and contradictory, especially in MCDA twin pregnancies after ART treatment. This may be explained by the rarity of MCDA twin pregnancies (2% of twin pregnancies) after ART treatment^5^. Importantly, the maternal complications in twin pregnancies from ART have not been analyzed with respect to chorionicity. MCDA twin pregnancies are at an increased risk of pregnancy-related complications compared to those with DCDA twins^12^,^13^,^14^. Obstetric outcomes of MCDA and DCDA twin pregnancies after ART vs spontaneous conception were thus respectively compared in the present study. Consistent with previous studies^8^, no significant difference in maternal outcomes is detected among DCDA twin gestations following ART compared to their corresponding spontaneous counterparts with exception of ICP the rate of which is significantly higher in DCDA pregnancies after ART in this study. However, the occurrence of maternal complications generally tends to be higher in MCDA pregnancies after ART compared to spontaneous cases. Even further, PPROM occurs in MCDA twin pregnancies following ART more than twice as frequently as natural MCDA twin pregnancies. Notably, similar to a recent report^16^, increased maternal age and heavier BMI are characterized in the cohort of MCDA twin pregnancies after ART in the present study, which may partially explain the higher prevalence of maternal complications. Residual confounding due to the underlying pathological conditions leading to infertility might at least be part of the explanation for no significance^15^. Unfortunately, a small sample size of 29 MCDA pregnancies after ART was included in this study, which may also preclude our findings reaching significance. A large, multicenter study may thus be required to investigate the ART–associated maternal risk, especially in MCDA twin pregnancies after ART.

Several studies shows the presence of two placental masses in DCDA twin pregnancies is associated with increased risk of maternal complications such as preeclampsia and GDM. This study also demonstrates an increased risk of preeclampsia, PPH and PPROM in DCDA twin pregnancies in the subgroup of twin pregnancies conceived spontaneously. However, this association was not detected in subgroup of twin pregnancies conceived following ART. Again, the rarity of MCDA twin pregnancies after ART limits the investigation on association between maternal risk and chorionicity in the subgroup of twin pregnancies conceived following ART in this study.

In our study, perinatal outcomes among MCDA twins after ART treatment were similar to those conceived spontaneously. This is in contrast to the study from Vandermeulen *et al.*, who reported that ART resulted in an excess of adversity in MCDA twins^17^. It is well recognized that perinatal outcomes in MCDA twins are primarily dependent on the presence or absence of the specific complications, such as TTTS^18^. Unfortunately, MCDA–specific complications were not differentiated from other outcomes in the cohort from Vandermeulen *et al.*, preventing us from further comparison. Similarly, we also found ART did not increase the risk of perinatal outcomes in DCDA twins. Our findings are in accordance with several previous reports^9^,^19^, but not all^6^,^8^. The disparities among these studies may be attributable to the different etiology of infertility, methods of ART, study design and sample size.

A number of studies report that MCDA twins have a higher perinatal risk than DCDA twins due to the ubiquitous vascular anastomoses in monochorionic placentas and associated complications. In the same line, this study showed that the perinatal risk in MCDA twins was significantly higher compared to DCDA twins, whether in the subgroup of spontaneous conception or ART. The present study further found MCDA twins are still at an increased risk of adverse perinatal outcomes compared to DCDA twins after excluding cases with anastomoses-associated complications such as TTTS, TAPS, TRAP and sIUGR of type II or III. Our findings are in agreement with a previous study from Leduc *et al.*^12^. However, Carter and Lewi *et al.* reported a comparable perinatal outcome between MCDA and DCDA twins with excluding specific complications for MCDA twins^18^,^20^. This may be due to the difference in prenatal monitoring and interventions at these centers^21^.

Several limitations of this study, besides its respective nature, should be considered. One is the small number of MCDA twin pregnancies after ART treatment prevents any firm conclusion, though a larger twin cohort was analyzed in this study compared to previous literature. This may be putative for a single–center study given the paucity of MCDA twin pregnancies (2%) following ART. Another limitation is that our findings may be confounded by the heterogeneity of infertility treated by ART. For instance, it is reported that the clinical expression after ART to treat blocked tubes in patients with younger maternal age and lower BMI is more favorable than that in patients developing ART due to other complications such as polycystic ovary syndrome^22^. However, it may be an arduous task to control for each type of infertility in a single–center study, even given the rarity of MCDA twin pregnancies after ART. Again, a well–controlled multicenter study is needed to study the impact of ART on clinical outcomes. Finally, iatrogenic damage to ova could occur during ART procedure. However, the extent to which ART procedure–related damage contributes to the adversity of perinatal outcomes could not be assessed in the present study.

In conclusion, based on the differentiation of chorionicity, the present study shows that IVF and/or ICSI is associated with a significantly increased the risk of PPROM in MCDA twin pregnancies and ICP in DCDA twin pregnancies. Perinatal outcomes in twin pregnancies after IVF and/or ICSI are similar to those conceived naturally.

## Materials and Methods

This was a retrospective review of all twin pregnancies undergoing serial ultrasound examinations and delivering at the Shanghai First Maternity and Infant Hospital from January 2010 to December 2014. This study was approved by the Shanghai First Maternity and Infant Hospital Scientific and Ethics Committees (registration number: 134119a4400). Written informed consent was obtained from each patient participating in the study. This study was performed in accordance with the approved guidelines without variations. A SPSS–based database was created for the registration of cases one by one. A research nurse was appointed to manage the database routinely following a tutorial providing definition for each variable. After the design of this study, the final version of the database was scrutinized by an experienced epidemiologist.

An ultrasound was performed between 7 and 14 weeks’ gestation to diagnose chorionicity. Chorionicity was confirmed by postnatal pathology. Discordance was corrected based on the pathology report. A second ultrasound for the anatomic survey was performed routinely. Twin pregnancies were excluded from this study if gross structural anomalies were detected given that those cases were managed with fetal reduction, feticide or termination of pregnancies. ART twin pregnancies were defined as those following *in vitro* fertilization (IVF) or IVF/intra-cytoplasmic sperm injection (ICSI).

Maternal obesity was defined as a Body Mass Index (BMI) of ≥30 kg/m^2^ at the first visiting to our center. Maternal complications were recorded, and included gestational hypertension, preeclampsia, gestational diabetes mellitus (GDM), intrahepatic cholestasis of pregnancies (ICP), placental previa, placental abruption, preterm premature rupture of membranes (PPROM) and postpartum hemorrhage (PPH). Gestational hypertension was defined as new onset of hypertension (systolic blood pressure ≥140 mmHg and/or diastolic blood pressure ≥90 mmHg) after 20 weeks of gestation without proteinuria; if proteinuria (>300 mg in 24 hours) was present, the diagnosis of preeclampsia was made. GDM was diagnosed based on the plasma glucose levels recommended by the National Diabetes Data Group in the most recent guideline from the American College of Obstetricians and Gynecologists^23^. Briefly, the plasma glucose threshold to diagnose GDM is 105 mg/dL for fasting blood test, 190 mg/dL at 1 hour after OGTT, 165 mg/dL at 2 hours after OCTT or 145 mg/dL at 3 hours after OCTT. The diagnosis of ICP was based on: 1) onset of generalized pruritus in the second or third trimester of pregnancy; 2) bile acid level >10 μmol/L; and 3) spontaneous relief within 3 weeks after delivery. Placenta previa was recorded according to the last ultrasound examination revealing the placenta covering internal os before delivery. PPROM was documented as rupture of membranes prior to 37 week’s gestation. Placental abruption was recorded if it was listed as a diagnosis on the medical record. PPH was defined as vaginal blood loss of ≥500 ml within 24 hours after delivery.

Adverse perinatal outcomes were recorded and included fetal loss (≤24 weeks of gestation), perinatal mortality (fetal demise after 24 weeks of gestation or neonatal death within 28 days after birth), delivery <28 weeks, delivery <32 weeks, birth weight, small for gestational age (SGA, defined as birth weight <the 10^th^ centile for gestational age^24^), birth weight <1500 gm, NICU admission and birth weight discordance (BWD)>25% calculated by the following formula: ((birth weight of larger twin–birth weight of smaller twin)/birth weight of larger twin) ×100.

Specific complications of MCDA twins were recorded and analyzed separately. These included twin–twin transfusion syndrome (TTTS), twin anemia–polycythemia sequence (TAPS), twin reversed arterial perfusion (TRAP) and selective intrauterine growth restriction (sIUGR). Diagnosis of TTTS was based on the Eurofetus criteria: polyhydramnios (deepest vertical pocket ≥8 cm before 20 weeks or 10 cm after 20 weeks of gestation) in the recipient sac and oligohydramnios (deepest vertical pocket ≤2 cm) in the donor sac^25^. Staging of TTTS was according to Quintero’s system^26^. TAPS was diagnosed according to the criteria proposed by Slaghekke *et al.*: Doppler ultrasound detecting an increase in middle cerebral artery peak systolic velocity (MCA-PSV) of over 1.5 multiples of the median (MoM) in one fetus that coincided with a decrease in MCA-PSV of less than 0.8 MoM in the co-twin; postnatal criteria consists of the hematology test (inter-twin Hb difference >8.0 g/dL and inter-twin reticulocyte count ratio >1.7) and placental injection showing only few small anastomoses present^27^. TRAP is defined as the blood flow pumped from the pump twin into the perfused twin. Diagnosis and classification of sIUGR were based on the Gratacos’s system^28^.

### Statistical Analysis

Kolmogorov-Smirnov test was used to examine the normality of data. Continuous data were analyzed using Student t-test or Mann-Whitney U test, where appropriate. Chi-square or Fisher’s exact test was employed to compare categorical variables. A p value of <0.05 was considered as statistical significance. A multiple logistic regression model was established to evaluate maternal complications and perinatal outcomes in relation to conception methods and chorionicity. The selection of independent variables for multiple regression model was based on the maternal characteristics which are significantly different between compared subgroups and are closely associated with pregnancy and perinatal outcomes.

## Additional Information

**How to cite this article**: Sun, L. *et al.* Clinical outcomes after assisted reproductive technology in twin pregnancies: chorionicity-based comparison. *Sci. Rep.* **6**, 26869; doi: 10.1038/srep26869 (2016).

## Supplementary Material

Supplementary Information

## Figures and Tables

**Table 1:** maternal characteristics by chorionicity and mode of conception.

	MCDA (n = 456)			DCDA (n = 697)		
	ART (n = 29)	SC (n = 427)	*P* value	ART (n = 382)	SC (n = 315)	*P* value
Maternal age–years^a^	32.9 ± 3.5	29.2 ± 3.9	<0.01	32.8 ± 3.5	30.4 ± 3.7	<0.01
Body mass index–kg/m^2 ^a	22.2 ± 3.9	20.6 ± 2.9	0.01	22.5 ± 3.1	22.5 ± 3.3	0.86
Multigravida–n (%)^b^	12 (41.4)	195 (45.7)	0.65	155 (40.8)	140 (44.4)	0.33
Nullipara–n (%)^b^	26 (90.0)	355 (83.1)	0.36	361 (94.5)	272 (86.3)	<0.01
Women with pre-existing diseases–n (%)^b^	4 (13.8)	35 (8.2)	0.30	25 (6.5)	33 (10.5)	0.06
Birth weight–g^a^	2169 ± 602	2177 ± 523	0.91	2463 ± 463	2478 ± 449	0.54

ART: assisted reproductive technology; SC: spontaneous conception.

^a^Student *t* test was used to compare continuous variables.

^b^Chi square test was used to analyze categorical variables. Pre-existing diseases included chronic hypertension, diabetes, thyroid disease, heart disease, lupus, asthma, HIV, hepatitis B and psychiatric disorders.

**Table 2:** Comparison of maternal complications by chorionicity and mode of conception.

	MCDA (n = 456)				DCDA (n = 697)			
	ART (n = 29)	SC (n = 427)	Unadjusted OR (95% CI)	Adjusted OR (95% CI)^a^	ART (n = 382)	SC (n = 315)	Unadjusted OR (95% CI)	Adjusted OR (95% CI)^b^
Gestational hypertension–n (%)	2 (6.9)	11 (2.6)	2.8 (0.6, 13.3)	4.3 (0.8, 24.4)	27 (7.1)	12 (3.8)	1.9 (1.0, 1.8)	1.5 (0.7, 1.6)
Preeclampsia–n (%)	2 (6.9)	23 (5.4)	1.3 (0.3, 5.8)	1.5 (0.3, 7.2)	40 (10.5)	31 (9.8)	1.1 (0.7, 1.9)	1.0 (0.6, 1.7)
GDM–n (%)	3 (10.3)	42 (9.8)	1.1 (0.3, 3.6)	0.5 (0.1, 2.4)	59 (15.5)	40 (12.7)	1.3 (0.8, 9.6)	1.0 (0.7, 8.4)
ICP–n (%)	1 (3.5)	13 (3.0)	1.1 (0.1, 9.0)	1.7 (0.2, 14.5)	27 (7.1)	6 (1.9)	3.9 (1.6, 7.2)	3.3 (1.3, 5.6)
Previa–n (%)	3 (10.3)	10 (2.3)	4.8 (1.2, 18.6)	4.5 (0.8, 24.6)	25 (6.5)	7 (2.2)	3.1 (1.3,18.3)	2.3 (0.9, 17.4)
Placental abruption–n (%)	0	0	–	–	2 (0.5)	1 (0.3)	1.7 (0.1, 1.5)	1.4 (0.1, 1.4)
PPH–n (%)	6 (20.7)	37 (8.7)	2.8 (1.1, 5.2)	2.4 (0.8, 8.3)	47 (12.3)	41 (13.0)	0.9 (0.6, 1.2)	0.9 (0.6, 1.4)
PPROM–n (%)	8 (27.6)	63 (14.8)	2.2 (0.9, 3.9)	3.0 (1.1, 3.2)	62 (16.2)	62 (19.7)	0.8 (0.5, 1.1)	0.9 (0.6, 1.2)
Maternal composite outcomes–n (%)	12 (41.4)	114(26.7)	1.9 (0.9, 4.2)	1.3 (0.5, 3.1)	182 (47.6)	121 (38.4)	1.5 (1.1, 2.0)	1.2 (0.8, 1.6)

GDM: gestational diabetes mellitus; ICP: intrahepatic cholestasis of pregnancies; PPH: Postpartum hemorrhage; PPROM: preterm premature rupture of membranes; ART: assisted reproductive technology; SC: spontaneous conception.

^a^Controlled for maternal age and body mass index.

^b^Controlled for maternal age and parity.

**Table 3:** Comparison of perinatal outcomes by chorionicity and mode of conception.

	MCDA (n = 456)				DCDA (n = 697)			
	ART (n = 29)	SC (n = 427)	Unadjusted OR (95% CI)	Adjusted OR (95% CI)^a^	ART (n = 382)	SC (n = 315)	Unadjusted OR (95% CI)	Adjusted OR (95% CI)^b^
Fetal loss–n (%)	4 (13.8)	46 (10.8)	1.3 (0.4, 4.0)	1.1 (0.3, 4.0)	6 (1.5)	2 (0.6)	4.2 (0.5, 35.8)	7.3 (0.8, 70.5)
Delivery < 28 wks–n (%)	5 (17.2)	58 (13.6)	1.3 (0.5, 3.6)	1.3 (0.3, 4.6)	4 (1.0)	3 (0.9)	1.1 (0.2, 5.0)	0.9 (0.2, 4.7)
Delivery < 32 wks–n (%)	8 (27.6)	94 (22.0)	1.3 (0.6, 3.1)	1.8 (0.7, 5.0)	21 (5.3)	22 (6.7)	0.7 (0.4, 1.4)	0.8 (0.4, 1.6)
BWD ≥ 25%–n (%)	5 (21.7)	61 (18.6)	1.2 (0.4, 3.4)	1.6 (0.4, 4.8)	33 (8.5)	25 (8.0)	1.0 (0.5, 1.7)	0.9 (0.5, 1.6)
Perinatal mortality–n (%)^c^	2 (3.5)	11 (1.3)	2.7 (0.6, 12.6)	2.5 (0.5, 12.6)	4 (13.8)	46 (10.8)	1.7 (0.1, 18.2)	2.6 (0.2, 32.9)
SGA–n (%)^c^	10 (20.8)	177 (26.0)	0.8 (0.4, 1.5)	0.9 (0.4, 2.0)	5 (17.2)	58 (13.6)	1.0 (0.8, 1.3)	1.1 (0.8, 1.4)
BW < 1500 gram–n (%)^c^	7 (12.1)	84 (9.8)	1.3 (0.6, 2.9)	1.7 (0.6, 4.5)	8 (27.6)	94 (22.0)	1.3 (0.7, 2.3)	1.4 (0.7, 2.5)
NICU admission–n (%)^c^	28 (70.0)	412 (68.0)	1.1 (0.5, 2.2)	1.5 (0.7, 3.4)	5 (21.7)	61 (18.6)	1.0 (0.8, 1.3)	1.0 (0.8, 1.3)

^a^Controlled for maternal age and body mass index.

^b^Controlled for maternal age and parity.

^c^Denotes the single infants instead of twin pair.Multiple logistic regression test was used to calculate the odds ratio (OR). BW: birth weight; BWD: birth weight discordance; SGA: small for gestational age; NICU: neonatal intensive care center. ART: assisted reproductive technology; SC: spontaneous conception

**Table 4:** Comparison of maternal complications between MCDA and DCDA pregnancies by control of conception model.

	Spontaneously–conceived twin pregnancies (n = 742)			ART–conceived twin pregnancies (n = 411)			
	MCDA (n = 427)	DCDA (n = 315)	Unadjusted OR (95% CI)	Adjusted OR (95% CI)^a^	MCDA (n = 29)	DCDA (n = 382)	OR (95% CI)^b^
Gestational hypertension–n (%)	11 (2.6)	12 (3.8)	1.5 (0.7, 3.4)	1.9 (0.7, 5.1)	2 (6.9)	27 (7.1)	1.0 (0.2, 4.5)
Preeclampsia–n (%)	23 (5.4)	31 (9.8)	1.9 (1.1, 3.4)	1.9 (1.0, 3.6)	2 (6.9)	40 (10.5)	1.6 (0.4, 6.9)
GDM–n (%)	42 (9.8)	40 (12.7)	1.3 (0.8, 2.1)	1.1 (0.6, 1.8)	3 (10.3)	59 (15.45)	1.6 (0.5, 5.4)
ICP–n (%)	13 (3)	6 (1.9)	0.6 (0.2, 1.6)	0.7 (0.2, 2.1)	1 (3.5)	27 (7.07)	2.1 (0.3, 16.3)
Previa–n (%)	10 (2.3)	7 (2.2)	0.9 (0.4, 2.5)	1.5 (0.5, 4.2)	3 (10.3)	25 (6.54)	0.6 (0.2, 2.1)
Placental abruption–n (%)	0 (0)	1 (0.3)	NA	NA	0 (0)	2 (0.52)	NA
PPH–n (%)	37 (8.7)	41 (13)	1.6 (1.0, 2.5)	1.8 (1.0, 3.0)	6 (20.7)	47 (12.3)	0.5 (0.2, 1.4)
PPROM–n (%)	63 (14.8)	62 (19.7)	1.4 (1.0, 2.1)	1.5 (1.0, 2.5)	8 (27.6)	62 (16.23)	1.0 (0.2, 4.5)
Maternal composite outcomes–n (%)	166 (38.9)	166 (52.7)	1.8 (1.3, 2.4)	1.7 (1.2, 2.4)	18 (62.1)	214 (56.02)	1.6 (0.4, 6.9)

NA: not applicable. GDM: gestational diabetes mellitus; ICP: intrahepatic cholestasis of pregnancies; PPH: Postpartum hemorrhage; PPROM: preterm previable rupture of membranes; ART: assisted reproductive technology.

^a^Controlled for maternal age and body mass index. Multiple logistic regression test was used to calculate the odds ratio (OR).

^b^No significant difference in maternal characteristics between the subgroup of MCDA and DCDA following ART. Univariate logistic regression test was used to calculate the OR.

**Table 5:** Comparison of perinatal outcomes between MCDA and DCDA pregnancies by control of conception model.

	Spontaneously–conceived twin pregnancies (n = 742)				ART–conceived twin pregnancies (n = 411)		
	MCDA (n = 427)	DCDA (n = 315)	Unadjusted OR (95% CI)	Adjusted OR (95% CI)^a^	MCDA (n = 29)	DCDA (n = 382)	OR (95% CI)^b^
Fetal loss–n (%)	46 (10.8)	1 (0.3)	0 (0, 0.2)	0.0 (0.0, 0.2)	4 (13.8)	5 (1.3)	0.1 (0, 0.3)
Delivery < 28 wks–n (%)	58 (13.6)	3 (1.0)	0.1 (0, 0.2)	0 (0, 0.2)	5 (17.2)	4 (1.1)	0.1 (0, 0.2)
Delivery < 32 wks–n (%)	94 (22.0)	20 (6.4)	0.2 (0.1, 0.4)	0.2 (0.1, 0.5)	8 (27.6)	18 (4.7)	0.1 (0.1, 0.3)
BWD ≥ 25%–n (%)	61 (18.6)	23 (7.7)	0.4 (0.2, 0.6)	0.4 (0.2, 0.7)	5 (21.7)	28 (7.5)	0.3 (0.1, 0.8)
Perinatal mortality–n (%)^c^	11 (1.3)	1 (0.2)	0.1 (0, 0.9)	0.1 (0, 0.9)	2 (3.5)	2 (0.3)	0.1 (0, 0.5)
SGA–n (%)^c^	177 (26.0)	115 (19.3)	0.7 (0.5, 0.9)	0.7 (0.5, 1.0)	10 (20.8)	143 (19.1)	0.9 (0.4, 1.8)
BW < 1500 g–n (%)^c^	84 (9.8)	20 (3.2)	0.3 (0.2, 0.5)	0.4 (0.2, 0.7)	7 (12.1)	31 (4.1)	0.3 (0.1, 0.7)
NICU admission–n (%)^c^	412 (68.0)	186 (32.8)	0.2 (0.2, 0.3)	0.2 (0.2, 0.3)	12 (30)	464 (67.4)	0.2 (0.1, 0.4)

BW: birth weight; BWD: birth weight discordance; SGA: small for gestational age; NICU: neonatal intensive care center. ART: assisted reproductive technology.

^a^Controlled for maternal age and body mass index. Multiple logistic regression test was used to calculate the odds ratio (OR).

^b^No significant difference in maternal characteristics between the subgroup of MCDA and DCDA following ART. Univariate logistic regression test was used to calculate the OR.

^c^Denotes the single infants instead of twin pair.

**Table 6:** Comparison of fetal outcomes by chorionicity and mode of conception: excluding TTTS, TAPS, TRAP and sIUGR of type II and III.

	ART (n = 404)			SC (n = 574)				
	MCDA (n = 22)	DCDA (n = 382) ref	Unadjusted OR (95% CI)	Adjusted OR (95% CI)^a^	MCDA (n = 259)	DCDA (n = 315) ref	Unadjusted OR (95% CI)	Adjusted OR (95% CI)^b^
Fetal loss–n (%)	0 (0)	5 (1.3)	–	–	7 (2.7)	1 (0.3)	8.7 (1.1, 71.3)	8.7 (1.1, 71.2)
Delivery < 28 wks–n (%)	3 (13.6)	4 (1.1)	14.9 (3.1, 71.5)	14.4 (2.9, 71.0)	11 (4.3)	3 (1.0)	4.6 (1.3, 16.7)	7.7 (0.9, 63.0)
Delivery < 32 wks – n (%)	5 (22.7)	18 (4.7)	5.9 (2.0, 17.9)	5.3 (1.7, 16.4)	23 (8.9)	20 (6.4)	1.4 (0.8, 2.7)	1.3 (0.6, 2.8)
BWD ≥ 25%–n (%)	3 (15.8)	28 (7.5)	2.3 (0.6, 8.4)	2.3 (0.6, 8.6)	16 (6.6)	23 (7.7)	0.9 (0.4, 1.6)	0.8 (0.4, 1.9)
Perinatal mortality–n (%)^c^	0 (0)	2 (0.3)	–	–	1 (0.2)	1 (0.2)	1.2 (0.1, 19.5)	0.9 (0.1, 14.8)
SGA–n (%)^c^	6 (15.8)	143 (19.1)	0.8 (0.3, 1.9)	0.8 (0.3, 1.9)	105 (21.6)	115 (19.3)	1.1 (0.9, 1.5)	1.1 (0.8, 1.5)
BW < 1500 gram–n (%)^c^	4 (9.1)	31 (4.1)	2.4 (0.8, 7.0)	2.0 (0.7, 6.2)	21 (4.1)	20 (3.2)	1.3 (0.7, 2.4)	0.9 (0.4, 2.1)
NICU admission–n (%)^c^	24 (72.7)	224 (32.6)	5.5 (2.5, 12.1)	5.2 (2.4, 11.5)	294 (63.4)	186 (32.8)	3.5 (2.7, 4.6)	3.3 (2.4, 4.4)

Multiple logistic regression test was used to calculate the odds ratio (OR). ART: assisted reproductive technology; SC: spontaneous conception.

^a^Controlled for maternal pre-existing diseases.

^b^Controlled for maternal BMI and age.

^c^Denotes the single infants instead of twin pair.

**Table 7:** Comparison of MCDA twin–specific complications between MCDA twin pregnancies conceived spontaneously and by ART.

	ART–Conceived MCDA twins (n = 29)	Spontaneous MCDA twins (n = 427)	P value
Twin–twin transfusion syndrome–n (%)	3 (10)	88 (21)	0.19
Twin anemia–polycythemia sequence–n (%)	0	4 (1)	0.78
Twin reversed arterial perfused sequence–n (%)	0	1 (0)	0.34
Selective intrauterine growth restriction of type II or III–n (%)	4 (14)	75 (18)	0.60

ART: assisted reproductive technology.

## References

[b1] Sutcliffe A. G. & Ludwig M. Outcome of assisted reproduction. Lancet 370, 351–359 (2007). 17662884 10.1016/S0140-6736(07)60456-5

[b2] Sun L. M. *et al.* Assisted reproductive technology and placenta-mediated adverse pregnancy outcomes. Obstet Gynecol 114, 818–824 (2009). 19888040 10.1097/AOG.0b013e3181b76bd1

[b3] Hansen M., Kurinczuk J. J., Bower C. & Webb S. The risk of major birth defects after intracytoplasmic sperm injection and *in vitro* fertilization. N Engl J Med 346, 725–730 (2002). 11882727 10.1056/NEJMoa010035

[b4] Schieve L. A. *et al.* Low and very low birth weight in infants conceived with use of assisted reproductive technology. N Engl J Med 346, 731–737 (2002). 11882728 10.1056/NEJMoa010806

[b5] Qin J. *et al.* Pregnancy-related complications and adverse pregnancy outcomes in multiple pregnancies resulting from assisted reproductive technology: a meta-analysis of cohort studies. Fertil Steril 103, 1492–508 (2015). 25910567 10.1016/j.fertnstert.2015.03.018

[b6] Caserta D. *et al.* Maternal and perinatal outcomes in spontaneous versus assisted conception twin pregnancies. Eur J Obstet Gynecol Reprod Biol 174, 64–69 (2014). 24405729 10.1016/j.ejogrb.2013.12.011

[b7] Daniel Y. *et al.* Analysis of 104 twin pregnancies conceived with assisted reproductive technologies and 193 spontaneously conceived twin pregnancies. Fertil Steril 74, 683–689 (2000). 11020507 10.1016/s0015-0282(00)01491-6

[b8] Moini A. *et al.* Obstetric and neonatal outcomes of twin pregnancies conceived by assisted reproductive technology compared with twin pregnancies conceived spontaneously: a prospective follow-up study. Eur J Obstet Gynecol Reprod Biol 165, 29–32 (2012). 22884795 10.1016/j.ejogrb.2012.07.008

[b9] Boulet S. L. *et al.* Perinatal outcomes of twin births conceived using assisted reproduction technology: a population-based study. Hum Reprod 23, 1941–1948 (2008). 18487216 10.1093/humrep/den169

[b10] Lynch A. *et al.* The contribution of assisted conception, chorionicity and other risk factors to very low birthweight in a twin cohort. Bjog 110, 405–410 (2003). 12699803

[b11] Wenstrom K. D., Syrop C. H., Hammitt D. G. & Van Voorhis B. J. Increased risk of monochorionic twinning associated with assisted reproduction. Fertil Steril 60, 510–514 (1993). 8375535 10.1016/s0015-0282(16)56169-x

[b12] Leduc L., Takser L. & Rinfret D. Persistance of adverse obstetric and neonatal outcomes in monochorionic twins after exclusion of disorders unique to monochorionic placentation. Am J Obstet Gynecol 193, 1670–1675 (2005). 16260208 10.1016/j.ajog.2005.04.007

[b13] Campbell D. M. & Templeton A. Maternal complications of twin pregnancy. Int J Gynaecol Obstet 84, 71–73 (2004). 14698833 10.1016/s0020-7292(03)00314-x

[b14] Oldenburg A. *et al.* Influence of chorionicity on perinatal outcome in a large cohort of Danish twin pregnancies. Ultrasound Obstet Gynecol 39, 69–74 (2012). 21830245 10.1002/uog.10057

[b15] Sibai B. Subfertility/infertility and assisted reproductive conception are independent risk factors for pre-eclampsia. Bjog 122, 923 (2014). 25288131 10.1111/1471-0528.13090

[b16] Simões T. *et al.* Outcome of monochorionic twins conceived by assisted reproduction. Fertil Steril 104, 629–632 (2015). 26093266 10.1016/j.fertnstert.2015.06.002

[b17] Vandermeulen L., Lewi L., DeKoninck P., Galjaard S. & Deprest J. 430: Monochorionic diamniotic twin pregnancies: outcome according to method of conception. Am J Obstet Gynecol 208, S188 (2013).

[b18] Lewi L. *et al.* The outcome of monochorionic diamniotic twin gestations in the era of invasive fetal therapy: a prospective cohort study. Am J Obstet Gynecol 199, 514.e1–8 (2008). 18533114 10.1016/j.ajog.2008.03.050

[b19] Li J., Yang J., Xu W. M., Cheng D. & Zou Y. J. Comparison of the perinatal outcome of twins conceived after assisted reproductive technologies versus those conceived naturally. J Reprod Med 60, 37–42 (2015). 25745749

[b20] Carter E. B., Bishop K. C., Goetzinger K. R., Tuuli M. G. & Cahill A. G. The Impact of Chorionicity on Maternal Pregnancy Outcomes. Am J Obstet Gynecol 213, 390.e1–7. (2015). 25986034 10.1016/j.ajog.2015.05.027

[b21] Ratha C. & Kaul A. OP31.03: Comparative outcomes in monochorionic and dichorionic twin pregnancies with active fetal surveillance and intervention. Ultrasound Obstet Gynecol 38, 146–146 (2011).

[b22] Beydoun H. A. *et al.* Polycystic Ovary Syndrome, Body Mass Index and Outcomes of Assisted Reproductive Technologies. Reprod Biomed Online 18, 856–863 (2009). 19490792 10.1016/s1472-6483(10)60037-5 PMC2744370

[b23] Practice Bulletin No. 137: Gestational diabetes mellitus. Obstet Gynecol 122, 406–416,(2013). 23969827 10.1097/01.AOG.0000433006.09219.f1

[b24] Lubchenco L. O., Hansman C., Dressler M. & Boyd E. Intrauterine growth as estimated from liveborn birth-weight data at 24 to 42 weeks of gestation. Pediatrics 32, 793–800 (1963). 14075621

[b25] Slaghekke F. *et al.* Fetoscopic laser coagulation of the vascular equator versus selective coagulation for twin-to-twin transfusion syndrome: an open-label randomised controlled trial. Lancet 383, 2144–2151 (2014). 24613024 10.1016/S0140-6736(13)62419-8

[b26] Quintero R. A. *et al.* Staging of twin-twin transfusion syndrome. J Perinatol 19, 550–555 (1999). 10645517 10.1038/sj.jp.7200292

[b27] Slaghekke F. *et al.* Twin anemia-polycythemia sequence: diagnostic criteria, classification, perinatal management and outcome. Fetal Diagn Ther 27, 181–190 (2010). 20339296 10.1159/000304512

[b28] Gratacos E. *et al.* A classification system for selective intrauterine growth restriction in monochorionic pregnancies according to umbilical artery Doppler flow in the smaller twin. Ultrasound Obstet Gynecol 30, 28–34 (2007). 17542039 10.1002/uog.4046

