# Supplementary material for: Clinical outcomes after assisted reproductive technology in twin pregnancies: chorionicity-based comparison
Source: Sci Rep. 2016 May 31;6:26869. doi: 10.1038/srep26869 (PMC4886640; doi:10.1038/srep26869)
Supplement: Supplementary Information [file srep26869-s1.doc]

**Clinical outcomes after assisted reproductive technology in twin pregnancies: chorionicity-based comparison**

Luming Sun^1^, Gang Zou^1^, Xing Wei^1^, Yan Chen^2^, Jun Zhang^2^, Nanette Okun^3^, Tao Duan^1*^

^1^ Fetal Medicine Unit & Prenatal Diagnosis Center, Department of Obstetrics, Shanghai First Maternity and Infant Hospital, Tongji University School of medicine , Shanghai, China

^2^ Xinhua Hospital, Shanghai Jiao Tong University School of Medicine, Shanghai, China

^3^ Maternal Fetal Medicine Program, Mt. Sinai Hospital, University of Toronto, Toronto, Ontario, Canada

Running title: clinical outcome of twin pregnancies after ART with respect to chorionicity

Corresponding author: Dr. Tao Duan, Department of Obstetrics, Shanghai First Maternity and Infant Hospital, Tongji University school of Medicine, 2699 Gao Ke West Road, Shanghai, 201204, China. Tel: [+86 21 5403 5206](javascript:void(0)); E-mail: [luming_sun@163.com](mailto:luming_sun@163.com)

Supplemental Table 1

|  | **MCDA (n=499)** | | | | **DCDA (n=729)** | | | |
| --- | --- | --- | --- | --- | --- | --- | --- | --- |
|  | **ART (n=34)** | **SC (n=465)** | **Interaction of maternal age and BMI** | | **ART (n=398)** | **SC (n=331)** | **Interaction of maternal age and parity** | |
|  |  |  | **F value** | **P value** |  |  | **F value** | **P value** |
| **Gestational hypertension – n (%)** | 3 (8.8) | 11 (2.4) | 1.12 | 0.35 | 27 (6.8) | 13 (3.9) | 1.33 | 0.26 |
| **Preeclampsia – n (%)** | 3 (8.8) | 24 (5.2) | 1.96 | 0.08 | 43 (10.8) | 36 (10.9) | 0.35 | 0.70 |
| **Gestational diabetes – n (%)** | 4 (11.8) | 45 (9.7) | 0.24 | 0.94 | 62 (15.6) | 43 (13.0) | 1.05 | 0.35 |
| **intrahepatic cholestasis of pregnancy – n (%)** | 1 (2.9) | 14 (3.0) | 1.25 | 0.29 | 29 (7.3) | 7 (2.1) | 0.24 | 0.78 |
| **Previa – n (%)** | 3 (8.8) | 11 (2.4) | 0.19 | 0.97 | 25 (6.3) | 7 (2.1) | 2.29 | 0.10 |
| **Placental abruption – n (%)** | — | — | — | — | 4 (1) | 1 (0.3) | 0.29 | 0.75 |
| **postpartum hemorrhage – n (%)** | 8 (23.5) | 38 (8.2) | 2.1 | 0.06 | 51 (12.8) | 44 (13.3) | 0.53 | 0.59 |
| **PPROM – n (%)** | 8 (23.5) | 64 (13.8) | 0.87 | 0.50 | 65 (16.3) | 70 (21.2) | 1.08 | 0.34 |

Data was displayed as n (%). ANOVA test was used to analyze the interactions of independent variables.

MCDA: monochorionic diamniotic twin pregnancy, DCDA: dichorionic diamniotic twin pregnancy, ART: assisted reproduction technology, SC: spontaneous conception, PPROM: preterm premature rupture of membranes.
